# The Alkaloidal Treatment of Pneumonia

**Published:** 1908-02

**Authors:** 


### The Alkaloidal Treatment of Pneumonia.

In patients with a full bounding pulse and a hot dry skin—sthenic cases—begin treatment with aconitine, veratrine and digitalin (Defervescent Comp. No. 1); or in patients having a small, quick, thready pulse and a hot dry skin—asthenic cases—give aconitine, digitalin and strychnine (Dosimetric Trinity No. 1). One granule of the combination selected should be given every 15 to 30 minutes until the pulse softens or the patient commences to sweat freely; then one every half hour to one hour as needed to maintain this effect. Keep the pulse at 80 or under if possible. Envelop the *entire* thorax (and from chin to low on hips) in a thin, closely-fitting jacket, thickly "quilted" with raw cotton, or the common "batten," well greased. If pain calls for treatment give a few doses of bryonin or hyoscyamine and codeine singly or in combination as indicated. Always secure complete defervescence and rest, no matter how much drug is required. Some cases do better on the above formulas alternated. Treatment should always be adjusted to it.

Clean out the prima viæ with 1-6-grain doses of calomel and podophyllin half hourly until one grain of each is taken; heaping teaspoonful of Saline Laxative in hot water and repeat every hour till bowels move freely; then give one or two 5-grain tablets (usually one) of the Compound Sulphocarbolates—Intestinal Antiseptics W-A—every two hours; or enough combined with occasional doses of Saline Laxative to keep the bowels sweet and clean. If stools are malodorous after they cease to be dark (almost black) in color, calcium sulphide granules, gr. 1-6 each (12 to 36 in divided doses daily) q. s. should be used as a systemic antiseptic, and nuclein should be pushed. Both intestinal and systemic antisepsis is of the utmost importance.

If seen early and properly selected remedies are pushed rapidly, nearly every case may be aborted. If the patient is naturally weak and has a rapid, thready pulse instead of a full, bounding pulse, always give strychnine arsenate in place of veratrine. Codeine may be used to quiet cough if required, and emetine to facilitate expectoration. Apomorphine in small doses for expectorant and relaxant effect often works best of all. If the pneumonic condition exists in a very young or very old patient and he does not cough and clear the bronchial tubes sufficiently, the stimulating expectorant, sanguinarine nitrate, should be used in just dose enough to produce sufficient coughing to rid the bronchial tubes of accumulated secretions. The complicating bronchitis may be as dangerous as the pneumonia itself. In such cases Calcidin (iodized calcium) is an exceedingly valuable remedy; furthermore, nothing can excel it for delayed resolution in any case. For the heart, strychnine has long been our sovereign remedy, but for heart waverings, in the earlier stages, we strongly advise the substitution of cactin, gr. 1-67 (one or more as needed), in place of strychnine. Later on, in cases foolishly allowed to run their course, the whip, strychnine, may be needed; and when rightly used will often prove a life saver.

It is of the greatest importance in all attacks of penumonia to have a hyperleukocytosis present. For the production and maintenance of this physiologic state, Nuclein, Abbott, is the greatest known remedy. In the milder cases give 20 drops three times a day on the tongue without water. In the more severe cases give 20 to 30 minims every 12 hours by hypodermic injection into deep muscular tissues. Nuclein not only produces hyperleukocytosis, but it acts as a powerful stimulant to every cell of the body.

In all cases leave the patient on strychnine or the Triple Arsenates with Nuclein, and occasional doses of cactin for the convalescent period, and continue the Saline Laxative and Intestinal Antiseptics that you have used throughout the case, q. s., as required.

The gist of the whole thing being: Forced defervescence, elimination, intestinal disinfection, systemic disinfection, local protection and strong support to nature's fighting forces.—Abbott, in *Clinical Medicine.*

